# Ten-year audit of clients presenting to a specialised service for young people experiencing or at increased risk for psychosis

**DOI:** 10.1186/s12888-014-0318-4

**Published:** 2014-11-18

**Authors:** Agatha M Conrad, Terry J Lewin, Ketrina A Sly, Ulrich Schall, Sean A Halpin, Mick Hunter, Vaughan J Carr

**Affiliations:** CTNMH (MH-READ), Hunter New England Mental Health and the University of Newcastle, McAuley Centre, The Mater, PO Box 833, Newcastle, NSW 2300 Australia; Schizophrenia Research Institute, Darlinghurst, Sydney NSW 2010 Australia; School of Psychiatry, University of New South Wales, Kensington, NSW 2033 Australia; Psychological Assistance Service, Hunter New England Mental Health, Newcastle, NSW 2300 Australia; School of Psychology, University of Newcastle, Callaghan, NSW 2308 Australia

**Keywords:** Psychosis, Risk status, Service evaluation, Comorbidity, Youth, Early intervention

## Abstract

**Background:**

Despite strong research interest in psychosis risk identification and the potential for early intervention, few papers have sought to document the implementation and evaluation of specialised psychosis related services. Assessment of Ultra High Risk (UHR) has been given priority, but it is equally as important to identify appropriate comparison groups and other baseline differences. This largely descriptive service evaluation paper focuses on the ‘baseline characteristics’ of referred clients (i.e., previously assessed characteristics or those identified within the first two months following service presentation).

**Methods:**

Data are reported from a 10-year layered service audit of all presentations to a ‘Psychological Assistance Service’ for young people (PAS, Newcastle, Australia). Baseline socio-demographic and clinical characteristics (N =1,997) are described (including clients’ psychosis and UHR status, previous service contacts, hospitalisation rates, and diagnostic and comorbidity profiles). Key groups are identified and comparisons made between clients who received ongoing treatment and those who were primarily assessed and referred elsewhere.

**Results:**

Clients averaged 19.2 (SD =4.5) years of age and 59% were male. One-tenth of clients (9.6%) were categorised as UHR, among whom there were relatively high rates of attenuated psychotic symptoms (69.1%), comorbid depression (62.3%), anxiety (42.9%), and attentional and related problems (67.5%). Overall, one-fifth (19.8%) experienced a recent psychotic episode, while a further 14.5% were categorised as having an existing psychosis (46.7% with a schizophrenia diagnosis), amongst whom there were relatively high rates of comorbid substance misuse (52.9%), psychosocial (70.2%) and physical health (37.7%) problems. The largest group presenting to PAS were those with non-psychotic disorders (43.7%), who provide a valuable comparison group against which to contrast the health trajectories of those with UHR and recent psychosis. Ongoing treatment by PAS was preferentially given to those experiencing or at risk for psychosis and those reporting greater current distress or dysfunction.

**Conclusions:**

Whether or not UHR clients transition to psychosis, they displayed high rates of comorbid depression and anxiety at service presentation, with half receiving ongoing treatment from PAS. Although international comparisons with similar services are difficult, the socio-demographic and comorbidity patterns observed here were viewed as largely consistent with those reported elsewhere.

## Background

### Identification of psychosis risk

For the past 25 years psychosis research among young people has tended to focus on identifying risk factors and providing options for early intervention for individuals in the initial stages of illness. The period to which the term ‘Ultra High Risk’ (UHR) relates is between the first noticeable changes in experience or behaviour and the appearance of overt psychotic symptoms [1]. The existing UHR criteria require that a young person (typically aged 14–30 years) who has been referred for mental health (MH) problems meets one or more of the following criteria: a) has experienced sub-threshold attenuated psychotic symptoms (APS); b) has experienced brief limited intermittent psychotic symptoms (BLIPS) – that is, episodes of psychotic symptoms that have lasted less than a week and resolved spontaneously; and/or c) meets trait and state risk factor criteria – having a first degree relative with a psychotic disorder or schizotypal personality disorder, whilst also experiencing a significant decline in functioning during the previous year [1-4]. Similar UHR criteria have been widely used throughout the world, together with various terms such as ‘clinical high risk’, ‘at risk mental state (ARMS)’, or ‘prodromal criteria’ [5,6]. As these terms are reasonably interchangeable, for the purposes of this paper, the term UHR will be used.

### Potential benefits and shortcomings from identifying psychosis risk status

While some centres and researchers have highlighted the importance of assessing UHR status, in order to provide early intervention, recent studies have also indicated that many individuals classified as UHR will not convert to psychosis, and equally, those who are deemed not at risk at the time of assessment may eventually develop the disorder [7-9]. For example, these findings have fuelled an ongoing debate in the *Australian and New Zealand Journal of Psychiatry* around the issue of establishing psychosis risk and the provision of additional early intervention services in Australia for people who are either at risk or have recently experienced their first episode [10-18].

Yung [12] defined early intervention as timely provision of professional help to an individual with a first episode of psychosis, arguing that much of the deterioration seen in clients with schizophrenia and other psychotic disorders is likely to be due to psychosocial factors that operate in the first few years of illness (as opposed to inherent biological processes). These factors include: disruption to education and career trajectory; family stress or breakdown; loss of friendships; depression; stigma; disruption of personality development; fear of relapse; substance misuse; homelessness; and post-traumatic stress disorder [12]. A recent comprehensive review of the psychosis risk literature [6] reinforces this view, demonstrating that clients at risk show psychopathologic, functional, neurocognitive and structural abnormalities. Additionally, another recent review of cognitive impairments and underlying pathophysiology [19] suggests that the early signs of structural and functional brain deficits among UHR clients are predictive of clinical outcomes. However, it is unclear to what extent these findings reflect a range of psychiatric disorders, psychological distress in general, or unique features of psychosis; hence, more longitudinal research is required comparing clients at risk who develop a psychotic disorder with those who do not.

Tanskanen and colleagues [20] conducted a recent qualitative study of help-seeking for a first psychosis episode, examining the perceptions of service users and carers. Common alternative explanations for psychotic symptoms included: substance misuse; stress; physical illness; and other psychological conditions [20]. These factors often add complexity to the clinical presentation and to the provision of early intervention. Yung [12] argues that provision of early intervention can either prevent these phenomena from occurring or minimise their impact on the individual. In addition, McGorry provides evidence from meta-analyses showing that early intervention reduces the risk of transition to psychosis to less than 10%, with psychological treatments such as cognitive behaviour therapy being as effective as antipsychotic medications [14,21].

A shorter duration of untreated psychosis (DUP) can reduce the initial psychosocial damage and the risk of traumatic forms of service entry and suicidal behaviours [22,23]. However, evidence for the efficacy of public health and educational campaigns in reducing DUP is mixed [24,25]. Comparable initiatives in Australia [26] and Canada [27] have not demonstrated the same benefits and further fuelled the debate about early intervention in psychosis. Castle [17] suggests that in Australia there is no good evidence that intervening early has any major effect on the longitudinal course of psychosis and certainly cannot prevent schizophrenia. However, regardless of whether clients meet UHR criteria, or are treated by specialist or generalist MH services, most are likely to present with sufficiently serious MH conditions to warrant treatment [15].

### Comorbidity considerations

There are high levels of comorbid drug and alcohol problems among individuals with schizophrenia and other psychotic disorders [28-30], as well as comorbid depression, dysthymia, and anxiety disorders [31], contributing to poorer global functioning and other negative outcomes [28]. Both alcohol and cannabis abuse/dependence among individuals with psychosis have been specifically associated with male gender and younger age [28-30].

Research suggests that the majority of clients presenting to services who meet UHR criteria in fact present with anxiety disorder or major depression [32-34]. Moreover, psychotic like experiences have also been linked with traumatic stress disorders and substance use/dependence [35]. Similarly, there is evidence of shared underlying features, such as altered cognitions, disturbed social and emotional functioning [36,37]. Additional support for common factors underlying both affective and psychotic pathology, or for reciprocal causal influence, comes from longitudinal studies showing subclinical psychotic experiences predict not only subsequent onset of psychotic disorders [38] but also affective disorders, even when psychotic experiences are not considered clinically relevant [39]. The co-occurrence of subclinical psychotic experiences and major depressive disorder also predict poorer outcomes [40]. Consequently, comorbidity issues need to be carefully considered amongst young people at risk for psychosis, as a potential predictor, moderator, or concurrent outcome.

### Limited reporting about implementation of specialised psychosis related services

Despite the relatively long-standing research emphasis on psychosis risk identification, and the potential for early intervention, there have been few published papers about the implementation and evaluation of associated specialised services [41-47]. Two Australian services previously documented are Melbourne’s Personal Assessment and Crisis Evaluation (PACE) clinic [42,44] and Newcastle’s Psychological Assistance Service (PAS, initial 2 years) [41]. When identifying individuals at risk, or within the early stages of a psychotic illness, most services have applied broadly similar criteria to the PACE clinic [3,42], but often with an additional focus (e.g., early prodromal and basic symptoms [45]; multi-element interventions for early psychosis [47]). Across these services, there have been consistently higher rates of presentations by males (60-70%), and amongst individuals meeting UHR criteria, attenuated symptoms have been more prominent. Referrals have typically been from MH services, with the particular pathways being largely determined by the health care systems within which those services operate (e.g., centralised public health systems in Australia and the United Kingdom, versus specialised and university clinics elsewhere).

### The current study

Although transition rates and the prediction of psychosis outcomes are still a central focus of research endeavour [48], there is a growing emphasis on the nature and severity of presenting problems, associated comorbidity, and both psychosis and non-psychosis outcomes [15,34,40]. Against this backdrop, and the limited publications reporting on the implementation of specialised psychosis related services, it is perhaps timely that we report the initial findings from our layered service audit (*Layer 1*: Baseline profiles).

The current service evaluation study was based on all presentations to a specialised ‘Psychological Assistance Service’ (PAS, Newcastle) for young people between service commencement in 1997 and December 2007; outcome data (relating to subsequent treatment, service contacts, admissions, transition to psychosis and comorbidity) were collected for a further two years, and will largely be the subject of future papers.

The primary aims of the present paper were threefold: 1) to describe the socio-demographic and clinical characteristics of PAS clients (including their psychosis and UHR status, previous service contacts, hospitalisation rates, and diagnostic and comorbidity profiles), and identify key groups for subsequent comparisons; 2) to document any service level or data collection factors or changes during the audit period that could impact on subsequent outcomes (and need to be considered as potential covariates); and 3) to examine the clinical and other characteristics of those clients who received ongoing treatment by PAS, relative to those who were primarily assessed and referred elsewhere.

Two problems that beset UHR and associated psychosis research relate to the identification of relevant comparison groups and the potential contamination/confounding of baseline and outcome assessments (e.g., where symptoms initially identified as transient at baseline actually continue until the first outcome assessment, and do not represent a new clinical episode or genuine transition). Many studies have contrasted UHR and first episode groups (e.g., [41,49,50]). While this is a valuable comparison for assessing underlying characteristics and possible developmental trajectories, it is less useful for examining differential conversion rates, since ‘*transition to psychosis*’ by those with UHR is essentially being compared with ‘*conversion to established psychosis*’ (generally schizophrenia) by those who already have a psychotic disorder. It is equally as important to examine subsequent transition to psychosis and associated characteristics and comorbidity by those who do not meet either psychosis or UHR criteria on initial presentation to services. By utilising all available service level data (as opposed to discrete assessments conducted solely for research purposes), the current study seeks to identify a clearer set of clinical groups (against which to compare subsequent clinical and service level outcomes for those classified as UHR), together with a better separation of baseline and outcome data.

## Methods

### The Psychological Assistance Service (PAS)

PAS was established in Newcastle (Australia) in 1997 and provided a community-based specialist service for young people (aged 12–25 years) who may have recently experienced a psychotic or related episode, or who were potentially at increased risk of developing a psychotic disorder. Psychosis risk status assessment was benchmarked against the criteria used by Melbourne’s PACE clinic [2,44], with PAS using comparable entry criteria and assessment methods (e.g., for assessing ‘at risk mental states (ARMS)’). Typically, this comprised a preliminary risk assessment followed by more intensive assessments conducted over multiple sessions (among those likely to be at increased risk, or with complex or unclear presentations), together with various neuropsychological and self-report measures [41]. Subsequently, PAS adopted a shorter assessment procedure that applied to all presentations, which was based on the PACE clinic’s Comprehensive Assessment of At Risk Mental States (CAARMS) [51]. The CAARMS instrument allows several aspects of risk to be coded, including family history, recent deterioration in functioning (e.g., a drop of 30 GAF points in the past 12 months), attenuated psychotic symptoms, and transient self-limited psychotic symptoms of less than one week duration (BLIPS). All (ARMS-based) assessments conducted prior to the introduction of the CAARMS were re-coded to an equivalent format. Approximately one-fifth of clients received ongoing (medium-term) treatment from PAS, which was provided by a small, multidisciplinary team of clinicians. This included psychiatric assessment and review, medication (if required), case management, cognitive behaviour therapy (CBT), family interviews, and/or other therapeutic interventions, as appropriate.

### Data sources

The data for this audit included both paper based and electronic clinical records from three different sources: 1) PAS clinical records; 2) community clinical records; and 3) hospital admission records. These data were processed separately initially, taking note of service delivery dates, and then any duplicate entries or inconsistencies were resolved. A PAS referral date was identified for each client, which was used as the basis for classifying all service occasions (i.e., community presentations or hospital admissions) into one of five timeframes: a) more than 2 years pre-PAS; b) up to 2 years pre-PAS; c) within two months following PAS referral (which we refer to as the two-month ‘PAS presentation window’); d) up to 2 years post-PAS (i.e., beyond the presentation window); and e) more than 2 years post-PAS.

Importantly, ‘baseline’ classification of clients into groups was based on all available service data up to and including two months post-PAS presentation. Any recorded psychosis episodes with dates before the PAS referral date were regarded as ‘existing psychosis’ episodes, while those falling (or assessed) within the PAS presentation window were regarded as ‘recent psychosis’ episodes. Although the term ‘first episode psychosis’ probably applies to many of the latter episodes, we have avoided using this term here because episode onset and frequency were not always assessed (only by exclusion) and a proportion of the ‘existing episodes’ would have also been ‘first episode psychosis’.

#### PAS data (presentations: 1997–2007, +2 years for ongoing treatment profiles and other outcomes)

Client information was initially translated from paper based service entry books into an electronic client database, which was subsequently matched against records from an existing PAS Access database. There were 2206 presentations to PAS during the audit period, 32 of which were excluded because of insufficient key identifying data (e.g., name or medical record number, referral or presentation dates). Among the remaining 2174 presentations, there were 1997 index presentations (by 1178 male and 819 female clients), with 177 repeat presentations (for separate clinical episodes involving the same clients).

On the basis of their own assessments (including the CAARMS), and other referral and service information, PAS initially classified all assessed clients in terms of their probable psychosis status and, where appropriate, their UHR status (including UHR subgroup status based on attenuated psychotic symptoms of recent onset, BLIPS, and/or trait-state risk factor criteria). The sole source of UHR status information was the PAS assessments, although (as described below) for the current analyses final assignment to groups was based on all available service level data.

#### Community data (1997–2009)

These data were extracted from two separate community sources: a) the ‘CROOS database’ (1997–2002), which was a stand-alone, MH service specific Access database containing limited client level information, service contacts and diagnoses; and b) the ‘CHIME system’ (2003–2009), an electronic database which progressively replaced CROOS from 2002–2003. CHIME is a comprehensive, community based client electronic record integrating data from community health and MH services. Service contacts, diagnostic data, and presenting problems/issues were initially processed separately; however, duplicate records and all admissions for persons younger than 12 years were deleted prior to the data being combined. As individuals varied in the length of time pre- and post-PAS for which service level data might be available (depending on their age at PAS presentation, the year in which they first presented, and the completeness of the relevant data systems), a common metric was adopted for calculating community contact rates, namely the number of service contact days per year (pro-rated for each individual based on the available data window). Similarly, to facilitate data aggregation, MH diagnoses and problems/issues were re-classified into nine common categories (psychosis, depression, anxiety, substance misuse, personality disorder, other MH problems, psychosocial issues, physical disorders, and other non-MH problems); when removing redundant records (e.g., equivalent diagnostic and problem/issue categories for the same service contact occasion) and for other hierarchical assignments, priority was given to diagnostic information (i.e., ICD-10 diagnoses) over ‘issues’ based CHIME listings.

#### Hospital admission records (1993–2009)

These data were extracted from two regional electronic hospital records systems: HOSPAS (1993–2003) and IPMS (2004–2009); only admissions from 12 years of age onwards were considered. Both systems collected comparable clinical data in terms of demographic information, length of stay, service use and diagnoses; any duplicate records were removed after data aggregation. Diagnoses were also re-classified into the nine common categories described above. Likewise, to adjust for variations in data availability timeframes across individuals, admission rates were calculated as admission days per year.

#### Ethics and data access approvals

As noted above, data reported here are from a layered service audit. Most aspects of this project received an exemption from formal review by the Hunter New England (HNE) Human Research Ethics Committee (letter dated March 25^th^ 2008), being viewed as part of an internal, low risk, service evaluation. Project layers involving linkages with other assessments conducted primarily for research purposes, and/or by research students, received separate approvals from the same Ethics Committee (03/12/10/3.16 and 12/11/21/5.06). Access to clinical records and selected databases within HNE MH services was approved by the Area Director of MH services, including access to PAS records and the CROOS database. Access to regional health databases (e.g., CHIME, HOSPAS, IPMS) was approved by relevant departmental managers from within HNE Local Health District. Project data management and analysis have been undertaken by the MH-READ unit within HNE MH services, of which the first three authors (AMC, TJL, KAS) are members.

### Allocation to baseline groups

The allocation of PAS clients (N =1997) to baseline clinical groups was *hierarchical* in nature, with priority given to any evidence of psychosis in the period up to and including the two-month PAS presentation window. This facilitated a more accurate classification of clients into five groups: Group A – ‘*existing psychosis*’ prior to the PAS referral date (based on all available evidence from community and admission records, PAS referral information and assessments); Group B – ‘*recent psychosis*’, based on similar information and assessments but with no evidence of psychosis prior to the referral date (only during the PAS presentation window); Group C – ‘*Ultra-High Risk (UHR)*’ group, based on PAS’s clinical assessment (and non-membership of groups A or B); Group D – ‘*non-psychosis MH disorders*’ group (e.g., depression, anxiety, substance misuse, personality disorder), based on all available service level data and assessments (and non-membership of groups A to C); and Group E – labelled as ‘*uncertain*’ (N =250 clients or 12.5%), about whom limited information was available (but with no basis for allocation to groups A to D), including individuals deemed not suitable for full assessment (13.9% of Group E), who refused assessment (13.4%), who were referred elsewhere (42.9%), or who had minimal or no contact with PAS (29.8%). Consequently, Group E was omitted from several of the analyses (e.g., when socio-demographic or diagnostic information was required).

### Statistical analyses

Microsoft Excel/Access and SPSS statistical software (Version 17.0; SPSS, Chicago, II, USA) were used for data processing and analyses. Chi-square tests (for categorical variables) and analyses of variance (for continuous variables) were used to examine differences between groups for the demographic variables, diagnostic and comorbidity profiles. In addition, community contacts and admissions were expressed as rates per year for the purposes of describing the data. Univariate and multivariate (two-step hierarchical) logistic regressions were used to examine predictors of treatment intensity within PAS. As a partial control for the number of statistical tests, the threshold for significance was set at p <0.01.

## Results

### Sample characteristics

Table 1 reports selected demographic and referral source information for the PAS clients included in the audit (N =1997). Overall, 14.5% were classified as having an ‘existing psychosis’ (Group A, N =289), 19.8% as ‘recent psychosis’ (Group B, N =395), 9.6% as UHR (Group C, N =191), and 43.7% as ‘non-psychosis MH disorders’ (Group D, N =872); while the remainder (12.5%) were assigned to the ‘uncertain’ group (Group E, N =250), having received minimal assessment and with limited or no information about previous contacts with health services. Within the UHR group, 26.2% reported a family history of psychosis (N =50), 69.1% had attenuated symptoms (N =132), and 16.2% had experienced BLIPS (N =31).

**Table 1:** **Baseline characteristics of clients presenting to PAS (N =1997)**

**Characteristic (%)**	**Overall**	**A: Existing psychosis (in pre-PAS period)**	**B: Recent psychosis (during PAS presentation window)**	**C: Ultra High Risk (UHR) group**	**D: Non-psychosis MH disorders**	**E: Uncertain**
	**(N =1997)**	**(N =289)**	**(N =395)**	**(N =191)**	**(N =872)**	**(N =250)**
Gender (% males)	59.0	68.5	62.8	57.1	56.3	52.8
Age						
Mean (SD)	19.2 (4.5)	21.0 (4.7)	19.6 (3.8)	17.6 (3.0)	18.9 (4.6)	19.1 (5.5)
Less than 18 years (%)	55.6	36.0	52.7	70.7	59.2	59.2
Referral source						
Self or family	18.4	5.9	12.2	16.8	23.1	28.0
Community MH	13.5	21.1	20.3	8.9	10.7	7.2
Hospital	14.8	42.9	15.4	6.3	10.1	4.4
GP	2.7	0.7	3.5	3.1	3.2	1.6
Other services	17.5	5.9	13.7	17.8	23.2	17.2
Unknown	33.0	23.5	34.9	47.1	29.8	41.6
Other demographic information available	53.6	46.4	71.9	67.5	44.6	-
**Characteristics of clients with available demographic data (%)**	**(N =936)**	**(N =134)**	**(N =284)**	**(N =129)**	**(N =368)**	
Marital status						
Single	92.9	92.5	92.6	96.9	92.0	
Educational level						
High school only	86.8	86.4	84.4	89.1	87.9	
Accommodation						
Live with family	77.9	81.2	78.9	81.3	74.9	
Live in house/flat	16.3	12.8	16.5	15.6	17.6	
Short term	5.8	6.0	4.6	3.1	7.5	
Employment status						
Employed	21.1	24.6	18.4	23.0	21.2	
Unemployed	43.8	57.5	46.3	27.8	42.4	
Student	35.2	17.9	35.3	49.2	36.4	

Overall there were more males (59.0%) who presented to PAS, with differential profiles across groups (χ^2^_(4)_ =20.0, p <0.001), associated with more males in the two psychosis groups. While the sample was relatively young, with an average age of 19.2 years, there were significant differences between groups (F_(4, 1997)_ =19.4, p <0.001), with the UHR group being the youngest (averaging 17.6 years). Referral source differences (χ^2^_(20)_ =369.2, p <0.001) followed a consistent pattern, with 42.9% of clients with existing psychosis referred by hospitals, 20.3% of clients with recent psychosis referred by community MH teams, and higher rates of self-referrals across the other groups (16.8% - 28.0%).

The lower portion of Table 1 summarises the other demographic data available for Groups A to D. There were no significant group differences for marital status, accommodation or employment, with the majority of clients being single (92.9%), living with family (77.9%), and having completed high school (86.6%). Employment differences (χ^2^_(6)_ =34.3, p <0.001) reflected the age profiles, with 49.2% of the UHR group being students.

### Psychosis profiles

Table 2 presents a breakdown of the psychosis related diagnostic profiles for the two psychosis groups, with individuals potentially allocated to multiple categories using all available information about psychosis episodes. The first seven categories in Table 2 are based on ICD-10 diagnoses (from hospital or community records), while the last three are based on psychosis related problems or issues identified in PAS clinical notes or CHIME electronic clinical records. Overall, the vast majority (92.7%) of those within the existing psychosis group had at least one formal psychosis diagnosis, including 46.7% with a schizophrenia diagnosis, 33.2% with an ‘unspecified nonorganic psychotic disorder’, 21.8% with an ‘acute or transient psychotic disorder’, and 20.4% with bipolar disorder (including mania); additionally, 38.8% also had at least one psychosis problem/issue identified in other clinical records. Among the recent psychosis group, the equivalent categories had generally lower rates, with 63.8% having a formal psychosis diagnosis (including 24.5% with schizophrenia and 7.1% with bipolar disorder); moreover, half (49.9%) of the recent psychosis group also had at least one psychosis problem/issue identified in other records.

**Table 2:** **Diagnostic profiles for psychosis groups**

**Diagnostic or problem category (%)**	**A: Existing psychosis (N =289)**	**B: Recent psychosis (N =395)**
Formal ICD-10 diagnoses	[92.7]	[63.8]
1. Schizophrenia	46.7	24.5
2. Persistent delusional disorder	8.0	2.0
3. Acute or transient psychotic disorder	21.8	22.0
4. Schizoaffective disorder	6.9	1.7
5. Other nonorganic psychotic disorder	1.4	0.7
6. Unspecified nonorganic psychotic disorder	33.2	21.5
7. Bipolar disorder (including mania)	20.4	7.1
Other clinical records (e.g., CHIME)	[38.8]	[49.9]
8. Schizophrenia (problem/issue)	11.1	18.9
9. Bipolar (problem/issue)	5.2	9.4
10. Other psychosis (problem/issue)	26.3	21.7

*Note*: Tabled values are the percentage of each group with any evidence of that diagnosis/problem during the time period, based on all available assessments (A: 698; B: 581), with assignments not mutually exclusive; aggregate psychosis rates for formal diagnoses and other clinical records are shown in square brackets; CHIME, electronic clinical record system used by community based services.

To further examine within-psychosis comorbidity, we pooled the formal ICD-10 diagnoses and the corresponding problems/issues identified in the other clinical records. For example, within the existing psychosis group, 52.9% (N =153) were identified as having schizophrenia and 23.2% (N =67) with bipolar disorder, with only a small overlap between these conditions (N =17, or 5.9%). Similarly, within the recent psychosis group, the corresponding aggregated rates were: schizophrenia, 41.5% (N =164); bipolar disorder, 15.7% (N =62); and overlap, 1.5% (N =6). Thus, the within-psychosis overlap between the schizophrenia and bipolar disorder categories was minimal.

In view of the ongoing debate within the clinical and research literature about what constitutes the prodromal phase of illness and transition to psychosis, we also examined the timing of psychosis episodes among clients allocated to the recent psychosis group. With respect to the first reported episodes within the PAS presentation window (N =395), half (57%) were identified from PAS clinical information, 9.9% came from inpatient records, and 33.2% from community records. Among the subset with detailed service delivery or assessment date information (N =298, 75.4%), the average time interval was 13.2 days post-PAS referral (with a median of 9 days); two-thirds (66.4%) of these assessments occurred within two weeks of PAS referral, while only 12.5% occurred more than four weeks after the referral date. The timing of these episodes (based solely on assessment dates) suggests that for the majority of clients the psychosis symptoms that were detected were probably also present at the PAS referral date and initial assessment rather than developing subsequently.

### Comorbidity profiles

As shown in Table 3, PAS clients had high levels of comorbidity across the various ‘baseline’ assessments and service presentations. Overall, there were significant group differences for every condition, except personality disorder. The highest levels of depression (62.3%) and anxiety (42.9%) were identified among the UHR group, while substance misuse (52.9%) and psychosocial issues (70.2%) were particularly notable among the existing psychosis group. The lower rate of substance misuse problems within the UHR group (6.8%) is not surprising, since 70.7% were under the age of 18 years at PAS presentation. Conversely, the relatively high rates of physical disorders (37.7%) and other non-MH problems (25.6%) among the existing psychosis group are noteworthy, considering that their mean age was only 21 years.

**Table 3:** **Baseline comorbidity profiles for PAS clients (N =1747)**

**Comorbid condition (%)**	**Overall (Excluding E: Uncertain group)**	**A: Existing psychosis**	**B: Recent psychosis**	**C:Ultra High Risk (UHR) group**	**D: Non-psychosis MH disorders**	**Statistical comparison (Groups A to D)**
	**(N =1747)**	**(N =289)**	**(N =395)**	**(N =191)**	**(N =872)**	
Depression	43.8	36.7	49.6	62.3	39.4	χ^2^_(3)_ =44.7**
Anxiety	25.1	21.5	34.2	42.9	18.3	χ^2^_(3)_ =72.7**
Substance misuse	26.5	52.9	37.0	6.8	17.3	χ^2^_(3)_ =201.7**
Personality disorder	17.1	16.6	16.2	20.9	16.7	χ^2^_(3)_ =2.3
Other MH problems^	67.3	68.9	55.7	67.5	72.0	χ^2^_(3)_ =33.3**
Psychosocial issues	40.4	70.2	39.2	41.9	30.7	χ^2^_(3)_ =141.1**
Physical disorders	14.0	37.7	13.2	6.3	8.3	χ^2^_(3)_ =168.3**
Other Non-MH problems	15.1	25.6	14.2	7.3	13.8	χ^2^_(3)_ =35.3**

*Note*: ^Within this category, the proportionate breakdown of conditions was: 70.1% attentional problems, 8.9% service related problems, 6.2% adjustment disorders, 5.6% childhood disorders, 1.6% intellectual disability, and 7.6% other problems (other behavioural and MH disorders, cognitive related problems, neurological disorders). Statistical significance: *p <0.01, **p <0.001.

### Service utilisation

Table 4 reports community contacts and hospital admission profiles for the five clinical groups. The first row of the table shows the estimated availability period for medical records, on which community contact and hospital admission rates were based; which, once again, partially reflects differences in the mean age across groups and variations across PAS presentation years. Overall, the PAS clients (N =1997) had 6.3 years of baseline data available, amounting to 9618 community contacts, or the equivalent of approximately one contact per client per year with any adult MH service. These rates are somewhat inflated by the high number of contacts within the short, two-month presentation window (which averaged over one contact per month). However, comparisons across groups are appropriate within the same metric; more generally, service contact rates are often inflated when sample selection is based on an index event (in this case, presentation to PAS). Those in the existing psychosis group had the highest aggregate contact rates per client per year (1.25), while the UHR and recent psychosis groups had the highest level of PAS contact during the presentation window (averaging 3.16 and 2.96 contacts, respectively). In relation to hospital admissions, 84.1% of the existing psychosis group had at least one MH admission, averaging 3.39 admission days per client per year, well above the other groups.

**Table 4:** **Baseline community contacts and admission profiles by PAS clients (N =1997)**

	**Overall**	**A: Existing psychosis**	**B: Recent psychosis**	**C: Ultra High Risk (UHR) group**	**D: Non-psychosis MH disorders**	**E: Uncertain**
	**(N =1997)**	**(N =289)**	**(N =395)**	**(N =191)**	**(N =872)**	**(N =250)**
Estimated availability of medical records - mean (median) years	6.3 (5.9)	7.7 (7.5)	6.6 (6.1)	5.4 (5.1)	6.2 (5.8)	5.5 (5.4)
Community contacts						
Total contacts	9618	2499	2253	980	3631	255
Average contact days per year (excluding presentation window)	0.98 (0.31)	1.25 (0.57)	0.99 (0.28)	1.38 (0.37)	0.99 (0.31)	0.33 (0.00)
Average number of PAS contacts within presentation window	2.25	2.32	2.96	3.16	2.07	1.01
All admissions (Min. age 12 years)						
Total admissions	1788	674	357	87	601	69
Total beddays	12748	6739	3042	297	2452	218
Any admission (%)	40.7	85.5	45.1	26.7	33.1	18.8
Average admission days per year	1.08	3.62	1.48	0.30	0.48	0.17
MH admissions						
Total admissions	1090	537	210	40	303	0
Total beddays	10787	6343	2438	214	1792	-
Any admission (%)	28.2	84.1	33.4	12.0	19.0	0.0
Average admission days per year	0.91	3.39	1.23	0.22	0.35	-

*Note*: The relevant timeframe includes the pre-PAS period plus the two-month PAS presentation window (i.e., presentations to youth and adult services, and admissions from the age of 12 years); average contact/admission days per year includes individuals with zero values; group E’s known contacts with MH services are largely limited to PAS.

**Figure 1:**
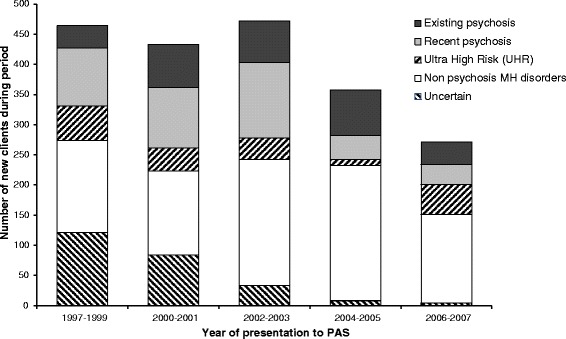
**Pattern of presentations to PAS by year.**

Figure 1 illustrates the pattern of presentations to PAS across the audit period. At least two trends are worthy of comment. Firstly, there was an overall reduction in the number of presentations from 2004 onwards, which coincided with service relocation, staffing alterations, and changes in referral and assessment procedures. Secondly, over time there was a proportionate reduction in the number of clients assigned to the uncertain group, which occurred for a mixture of reasons: improved access to electronic records; improvements in clinical documentation and assessment procedures (including the change to a more uniform, CAARMS based assessment); and the simple increased volume of potential pre-PAS records per client as time advances (given the fixed starting dates for the available electronic databases – 1993 for hospital records and 1997 for community records).

### Potential confounders

Based on the service changes noted above, the various data collection methods and changes across the audit period, and the preliminary analyses, a small set of six potential covariates was selected (that could impact on subsequent outcomes): gender (male: N =1178; female: N =819); age group (under 18 years: N =1111; 18+ years: N =886); service year block (1997–2003: N =1369; 2004–2007: N =628); PAS treatment status (assessment only: N =1593; some ongoing treatment: N =404); and the length of potential data availability windows per client (pre-PAS plus presentation window: mean =6.32, SD =2.82, range =0.17 to 14.93 years; post-PAS: mean =7.40, SD =2.86, range =1.86 to 12.76 years).

### PAS treatment status

The uncertain group (Group E) was excluded from the analysis of PAS treatment status, due to the absence of information about comorbidity, leaving N =1747 clients in this analysis. During the first 26 months after PAS referral (i.e., the presentation window plus 2 years), the average number of PAS contacts was 4.86 (SD =6.89, median =2, range 1 to 64); three-quarters (76.4%) of which occurred within 8 months of PAS referral. For convenience, we identified two sub-groups: those who primarily received an assessment (i.e., with 6 or fewer contacts; N =1342, mean =2.11, median =1 contact); and those receiving some ongoing treatment (N =404, or 23.1%, mean =13.98, median =10 contacts). The outcome variable for the logistic regression analyses was treatment status (0: assessment only *vs*. 1: ongoing treatment), the other five potential confounders listed above were included as covariates in the multivariate analysis (at step 1), and there were nine main predictors (at step 2): clinical group, with non-psychosis MH disorders (Group D) as the reference group, and the eight baseline comorbidity variables identified in Table 3 (categorized as 0: absent *vs*. 1: present).

As shown in Table 5, relative to those with non-psychosis MH disorders (12.3% receiving PAS treatment), there were significantly higher (p <0.001) rates of ongoing treatment among the existing psychosis (25.6%, Adjusted Odds Ratio, AOR =3.40), recent psychosis (33.4%, AOR =5.80) and UHR groups (47.6%, AOR =9.57). In addition, higher ongoing treatment rates were experienced by those with depression (28.5%, AOR =1.54, p =0.002) or anxiety (33.7%, AOR =1.62, p =0.002), and lower rates by those with substance misuse (17.3%, AOR =0.53). There was also a tendency for those with other comorbid MH problems to have higher treatment rates (28.1%, AOR =1.59, p =0.010). Psychosocial issues, physical disorders and other non-MH problems were not significantly associated with treatment by PAS. In short, PAS was more likely to provide treatment to those at risk for or experiencing psychosis and to those with greater distress (e.g., anxiety/depression) or dysfunction (e.g., attentional problems), a treatment pattern that is consistent with the service’s broad specialisation.

**Table 5:** **Relationships between baseline client characteristics and receipt of ongoing treatment by PAS (N =1747)**

**Baseline characteristic**	**% Receiving ongoing PAS treatment**	**Unadjusted Odds Ratio (OR)**	**99% CI**	**Adjusted Odds Ratio (AOR)**	**99% CI**
Gender: Male	23.2 (*vs.* 23.0)	0.98	(0.73, 1.32)	0.89	(0.64, 1.26)
Age: Less than 18 years	26.1 (*vs.* 19.5)	1.45**	(1.07, 1.96)	1.05	(0.59, 1.87)
Diagnostic group					
Existing psychosis	25.6	2.46**	(1.59, 3.81)	3.40**	(2.11, 5.48)
Recent psychosis	33.4	3.58**	(2.44, 5.25)	5.80**	(3.75, 8.96)
Ultra high risk	47.6	6.50**	(4.11, 10.28)	9.57**	(5.66, 16.18)
Non-psychosis MH disorders	12.3 (ref)	1.00		1.00	
Comorbid condition^¥^					
Depression	28.5 (*vs.* 18.9)	1.70**	(1.27, 2.28)	1.54*	(1.07, 2.22)
Anxiety	33.7 (*vs.* 19.6)	2.09**	(1.52, 2.86)	1.62*	(1.09, 2.39)
Substance misuse	17.3 (*vs.* 25.2)	0.61**	(0.43, 0.88)	0.53**	(0.33, 0.84)
Personality disorder	23.5 (*vs.* 23.1)	1.02	(0.69, 1.50)	0.87	(0.54, 1.39)
Other MH problems^	28.1 (*vs.* 12.8)	2.67**	(1.85, 3.84)	1.59*	(1.00, 2.54)
Psychosocial issues	22.1 (*vs.* 23.8)	0.90	(0.67, 1.22)	0.81	(0.54, 1.21)
Physical disorders	29.0 (*vs.* 22.2)	1.43	(0.96, 2.12)	1.20	(0.70, 2.03)
Other Non-MH problems	22.3 (*vs.* 23.3)	0.94	(0.62, 1.43)	0.66	(0.39, 1.11)

*Note*: Significance tests were based on Wald statistics: *p <0.01, **p <0.001; adjusted analyses controlled for all tabled variables, plus service year block and available data windows (pre-PAS ). ^¥^Includes history of comorbid conditions or concurrent conditions present at the time of assessment. ^See Table 3 for further details.

## Discussion

### Who presented to PAS

PAS in Newcastle and the PACE outpatient clinic in Melbourne [2] were deliberately established with generic names, to reduce potential stigma and to encourage young people experiencing a range of difficulties to attend. More recently, similar services around the world (and those linked to research programs, in particular) have tended to use more selective referral networks and screening processes to identify a narrower range of potential clients. For the problems that comprised the primary PAS target groups, the yield across the audit period was considered reasonable (existing/recent psychosis yield: 684/1997 or 34.3%; UHR yield: 191/1997 or 9.6%). However, compared to the other psychosis-related services described earlier, PAS had a much higher percentage of ostensibly non-psychosis related presentations (872/1997 or 43.7%), largely reflecting differences in health care systems and referral/assessment processes; which, on the positive side, provided a naturalistic comparison group for examining future transition to psychosis. PAS also developed a local reputation for undertaking comprehensive diagnostic assessments, which may have accounted for some referrals of complex cases (with or without a possible psychosis).

There was a higher overall percentage of males (59%) presenting to PAS, which is consistent with similar services elsewhere (e.g., [45]), and the majority of clients (55.6%) were less than 18 years of age. Two-thirds of clients (68.5%) with an existing psychosis were male, and they also experienced the highest rates of comorbid substance misuse (52.9%), psychosocial (70.2%), physical health (37.7%) and other non-MH (25.6%) problems, and MH admissions (84.1%); these findings are consistent with the profiles highlighted in recent Australian National MH Report Cards [52] for individuals with an established serious mental illness. With respect to diagnosis, almost half (46.7%) of those with existing psychosis received a schizophrenia diagnosis, compared with one-quarter (24.5%) of the recent psychosis group; this pattern probably reflects a mixture of illness stage differences (e.g., evidenced by different comorbidity profiles – see Table 3) and data source differences (e.g., diagnostic/assessment practice differences associated with variations in the rates of previous hospital admissions and subsequent referrals – see Tables 1 and 4). Notwithstanding, subsequent conversion rates to schizophrenia amongst the other members of the recent psychosis group (i.e., not presently identified as having a schizophrenia diagnosis or problem) could be a worthwhile additional component in future analyses.

Among the UHR group, half of whom (49.2%) were students, there were high rates of attenuated psychotic symptoms (69.1%), in accord with previous studies (e.g., [45,53]); likewise, the BLIPS rate (16.2%) was approximately midway between the older and more recent naturalistic studies reviewed by Simon and colleagues [9]. There were also relatively high rates of comorbid depression (62.3%), anxiety (42.9%), and attentional and related problems (67.5%), which may have precipitated service presentation at that time; indeed, the majority of this group’s baseline community contacts (604/980 or 61.6%) with MH services were with PAS. Comorbidity profiles among the UHR group were also generally comparable to those reported elsewhere [32-34], with the low rate of substance misuse problems (6.8%) consistent with their mean age of 17.6 years. While such problems typically go beyond UHR criteria, they are the focus of everyday clinical attention and contribute to the reasons for clients seeing help [5]. Ongoing monitoring of risk, and any signs of deterioration, can be undertaken while simultaneously addressing these presenting problems [5].

Reductions in annual presentations to PAS and in the percentages of clients assigned to the ‘uncertain’ category (see Figure 1) are reflective of service, assessment, and data accessibility changes over time, and not an underlying change in the relevant regional population. However, such changes, together with the observed baseline differences between the clinical groups in socio-demographic characteristics (Table 1), comorbidity profiles (Table 3), and involvement in ongoing PAS treatment (Table 5), highlight the fact that these potential confounders need to be carefully taken into account in future analyses of PAS outcomes (such as comparisons between groups in transition rates and subsequent comorbidity and service utilisation).

### Who received treatment

Several factors are likely to have contributed to PAS treatment patterns. Firstly, the service’s major focus on those at risk for or experiencing psychosis is reflected in the higher percentages receiving ongoing treatment within the UHR (47.6%) and recent psychosis (33.4%) groups. Secondly, the observed associations between higher ongoing treatment rates and comorbid depression/anxiety and other MH problems, but not substance misuse, may partially reflect the nature of the psychological treatments likely to be offered (such as CBT), relative to other specialised services to which PAS could refer (e.g., substance misuse services), together with specific concerns for clients experiencing greater distress or dysfunction at presentation.

Those receiving ongoing PAS treatment averaged approximately one session every 8 weeks during the first two years post-presentation; however, in practice, more intensive treatment was delivered during the first six months, followed by periodic review. Having contact with PAS as part of an early intervention strategy might have been beneficial to these clients, especially with respect to education about mental illness, reducing stigma, or providing diagnostic information. In any event, on average, clients who received ongoing PAS treatment appear to have had either more complex or more symptomatic presentations.

As noted earlier, in some instances clients were seen by PAS primarily for the clarification of diagnostic issues, which would be expected to occur more frequently among those displaying comorbidity. Further, research evidence suggests that symptoms of depression and anxiety are present in the majority of clients with schizophrenia, and perhaps these symptoms could be used to identify distinct clinical subgroups among those with psychosis [40]. Moreover, recent studies have shown that anxiety disorders, particularly social anxiety, are highly prevalent and contribute significantly to a reduction in quality of life, particularly social isolation, feelings of helplessness, and lower self-esteem [31,35].

### Future directions

The current paper is largely descriptive in nature and we acknowledge that it is difficult to directly compare psychosis related services operating in different countries and within different health care models, which may partially account for the limited number of published papers documenting the implementation and evaluation of such services. Notwithstanding, we encourage others to undertake similar comprehensive service audits, primarily because they assist in the review and refinement of services, but also because they stimulate consideration and investigation of related research, treatment and translational issues. Such evaluations also need to both inform and be mindful of strategic changes in health service delivery for young people that are currently being undertaken in many countries (e.g., *eHealth* based initiatives). In Australia, for example, a range of ‘*headspace* centres’ have now been established, designed as “*highly accessible, youth-friendly, integrated service hubs that provide evidence-based interventions and support to young people aged 12–25 years around their mental health, health and wellbeing needs*” [54].

It may be an obvious methodological observation, but it is worth noting again that different patterns of comparisons between clinical groups, ideally across multiple time points, can provide different insights about the underlying problems. So, for example, comparisons within UHR groups between those experiencing and those not experiencing a certain outcome, such as transition to psychosis, or UHR remission [9], can aid our understanding of potential mediators and/or contextual or environmental factors at play; while comparisons between UHR and first episode groups can potentially help to identify some of the prerequisite conditions or early changes associated with the disorder of interest. In the current service evaluation project, there is a further opportunity to utilise a relatively novel comparison group, those with non-psychotic MH disorders (and non-UHR) at baseline, the largest subgroup presenting to PAS. This will enable us to explore questions in future papers such as the following: do UHR individuals have differential psychosis transition rates, or experience different comorbidity or service outcomes, compared to those with similar socio-demographic characteristics who presented to the same services during the same time period, but amongst whom neither psychosis nor associated risk was evident?

### Strengths and limitations

While the current dataset provides a basis for comparing several groups of young clients, who are presumably at various illness stages, it nevertheless draws from initial presentations to a single Australian psychosis related service. There are both advantages and disadvantages to using real-world service level data, as opposed to data collected within a formal research framework, which largely boil down to breadth versus depth issues. Service level data is particularly useful for accessing accurate information about service contacts, admissions, presenting problems, and so on, over extended periods of time, relative to self-reported research measures. Larger sample sizes, a lower likelihood of selection biases (e.g., by considering all presentations), and minimal exclusion criteria (e.g., retention of those not meeting UHR criteria), are inherent strengths of service level data.

However, the quality and availability of service level data can be variable. In our case, we used all available baseline service level data for a period exceeding a decade; moreover, for each individual, we essentially looked beyond a single assessment timepoint to a service based window in time (for many clients, from approximately 12+ years of age to service presentation). On the other hand, research studies typically use standardised scales and structured assessments to more comprehensively assess clinical characteristics, risk factors, treatment fidelity, and selected outcomes. For example, within the current project, one of the obvious limitations was the lack of access to detailed comparative information about current symptomatology, illness chronicity, specific treatments received, or medication usage.

## Conclusions

We have successfully extracted and processed a large amount of service level data (from multiple sources) relating to presentations over a decade to a specialised service for young people experiencing or at increased risk for psychosis. This has facilitated the identification of two broad groups – those who have already experienced at least one episode of psychosis (N =684, sub-categorised as existing or recent psychosis) and those without any such episodes, but who vary in their assessed levels of risk for psychosis (N =1313, sub-categorised as UHR, non-psychosis MH disorders, or uncertain). Observed differences between these groups were largely consistent with expectations based on the research literature (e.g., a high percentage of males; moderate to high levels of comorbidity, especially amongst those with existing psychosis; and high rates of attenuated psychotic symptoms, depression and anxiety among the UHR group). Unlike other specialised psychosis-related services, a substantial percentage of PAS presentations displayed neither a psychotic disorder nor an increased risk. It is anticipated that this sub-group will provide a valuable reference point for future analyses, against which to contrast the health trajectories of the UHR and recent psychosis groups in particular.

## Abbreviations

AOR: Adjusted odds ratio
APS: Attenuated psychotic symptoms
RMS: At risk mental state
BLIPS: Brief limited intermittent psychotic symptoms
CAARMS: Comprehensive Assessment of At Risk Mental States
CBT: Cognitive behaviour therapy
CHIME: Clinical information and management exchange
CROOS: Community register of occasions of service
DSM-IV: Diagnostic and Statistical Manual of Mental Disorders – Version IV
DUP: Duration of untreated psychosis
GAF: Global assessment of functioning
HOSPAS: Hospital patient administration system
HNE: Hunter New England (health district, Australia)
ICD-10: International Classification of Diseases (Version 10)
IPMS: Inpatient management system
MH: Mental Health
MH-READ: Research Evaluation Analysis and Dissemination unit within local MH service
PACE: Personal Assessment and Crisis Evaluation clinic (Melbourne, Australia)
PAS: Psychological Assistance Service (Newcastle, Australia)
SD: Standard deviation
SPSS: Statistical Package for Social Sciences
UHR: Ultra High Risk (for psychosis)
